# Influence of Processing and Mix Design Factors on the Water Demand and Strength of Concrete with Recycled Concrete Fines

**DOI:** 10.3390/ma19020237

**Published:** 2026-01-07

**Authors:** Leonid Dvorkin, Vadim Zhitkovsky, Nataliya Lushnikova, Vladyslav Rudoi

**Affiliations:** 1 Department of Building Elements Technology and Materials Science, National University of Water and Environmental Engineering, 33028 Rivne, Ukraine; v.v.zhitkovsky@nuwm.edu.ua (V.Z.); v.v.rudoj@nuwm.edu.ua (V.R.); 2 Building Materials Group, Department of Built Environment, Eindhoven University of Technology, Groene Loper 3, 5612 AE Eindhoven, The Netherlands; n.lushnikova@tue.nl

**Keywords:** recycled concrete fines, concrete, wastes, strength, experiment planning, model

## Abstract

The study examines how crushed and sieved concrete rubble—recycled concrete fines (RCF) and the ways of their reactivity activation—affect processing, mix design, and properties of cement-based concrete. Based on the relationship to mass loss during crushing, the compressive strength of the concrete fines processed from rubble was initially determined. The morphology of the particles as well as the chemical and mineralogical composition of RCF were ascertained using XRD, SEM, and EDS characterization tests. Certain RCF surface area (fineness) and type of treatment are associated with specific pozzolanic activity of RCF. Using the approaches of factorial experimental design, tests were planned by varying six factors: RCF specific surface area, RCF content, thermal treatment temperature of RCF, cement content, superplasticizer dosage, and hardening accelerator (Na_2_SiF_6_) content in concrete containing RCF. Statistical processing of the research results data provided adequate polynomial regression models for the water demand of the concrete and the compressive strength of hardened concrete at 7 and 28 days. The models were quantitatively analyzed to evaluate the influence of the studied factors on the output parameters and to rank them according to their impact. The greatest increase in water demand was attributed to cement content change, in particular above 400 kg/m^3^, and to RCF content. It was established that the addition of a superplasticizer compensated for additional water demand and the reduction in compressive strength caused by partial replacement of cement with RCF. Increasing the specific surface area of RCF up to a specific surface area of 250 m^2^/kg improved compressive strength but further grinding caused strength reduction due to increased water demand. The positive effect of the superplasticizer on RCF-modified concrete strength was enhanced by the introduction of a chemical activator (hardening accelerator) and thermal treatment of RCF. The obtained models of water demand and compressive strength of concrete with RCF can be applied for the optimization of the mix design. This paper proposes a method of mix design and provides an example of calculation.

## 1. Introduction

Enhancing both the economic and environmental performance of concrete has become a critical priority in the construction sector nowadays. As is well known, the production of Portland cement (PC) requires significant energy consumption and is accompanied by a large carbon footprint, which has a high impact on climate change [1]. Reduction in cement consumption can be achieved by a set of technological measures, the most common of which is the partial replacement of PC with mineral additives. The range of mineral additives used in the production of both cement and concrete is quite wide, with granulated blast-furnace slags and fly ash being the most demanded. However, by 2050, it is expected that blast-furnace slags and fly ash will be able to meet only 20% of the global demand for cement production; therefore, expanding the range of active mineral additives is a pressing issue [2,3]. Therefore, there is a need for alternative high-volume sources of mineral admixtures, preferably secondary materials, that are environmentally beneficial.

Among the materials that require recycling are construction and demolition wastes (CDW), disaster and war conflict debris rubble, the volume of which is constantly increasing in the world. According to the Waste Framework Directive, the EU generates 700 mln. t of CDW annually [4]. It is prognosed that China will generate 224.08 billion tonnes (Bt) of CDW from 2000 to 2100, mostly gravel (34.15%), sand (30.08%), and brick/tile (14.37%) [5]. The composition of CDW and especially debris is diverse, but it usually contains predominantly cement-based concrete and brick demolition debris (up to 80%). In Ukraine, the intensive increase in the volume of war debris is caused by the destruction of buildings and structures as a result of military actions. According to official data, there were more than 600 thousand tons of debris in Ukraine by June 2024. However, this number may be significantly underestimated [6].

A significant number of studies on the use of recycled concrete have already been conducted [7,8,9]. One of the key research directions is the use of recycled concrete fines (RCF) as an active mineral additive in concrete [10].

As is known, the effect of active mineral additives on the properties of cement-based concrete is determined by chemical and physicochemical processes that occur during their structure formation and hardening [11]. Due to the pozzolanic activity of additives, hydration products are synthesized in the cement matrix, which makes reduction in cement consumption possible without a significant change in the concrete’s basic properties. It has been experimentally established [12,13,14,15,16] that recycled concrete fines have a certain pozzolanic activity, which depends on their composition and specific surface area. The chemical activity of concrete fines is also complemented by its ability to hydrate unreacted cement particles, which depends on the cement content in the original concrete, as well as on the curing time and conditions.

Having a high specific surface area, concrete fines also affect physicochemical processes at the interfacial transition zone (ITZ). According to the nucleation (Gibbs–Volmer) theory [17], they can serve as crystallization centers and promote the formation of crystal nuclei of new hydration products. Based on the Gibbs–Volmer theoretical calculations, thermal treatment and decreasing particle size enhance the crystallization behavior of cement systems containing dispersed fillers. The formation of the concrete structure is also significantly influenced by so-called ‘constrained conditions’ [18], which are characterized by a sharp increase in the solid phase when introducing active fillers and forming water films at the surface of the mineral admixtures.

An important parameter affecting the properties of concretes is the strength of the interfacial transition zone between the binder and aggregates. In the study [11], it was shown that reducing the intergranular distances in mortars on quartz sand from 210 to 30 nm leads to increasing the hardness of the cement pastes. Ensuring the optimal thickness of the interfacial layer when introducing mineral additives requires a sufficiently high specific surface and the optimal content of the additive. Strong adhesion contacts in the cement–additive system can be explained by the higher surface energy of the additive (adhesive) than that of the cement. This conclusion is based on the thermodynamic concept of adhesion, according to which the surface energy of the adhesive should be higher than that of the substrate [18]. The increase in surface energy of concrete fines is achieved by breaking intermolecular bonds in their structure during grinding. This process leads to surface amorphization, an increase in the isobaric potential of fines, and consequently, to higher chemical reactivity.

A significant positive effect was observed when active mineral additives were added together with surface-active substances (SAS) [19]. A necessary condition for the effectiveness of SAS is their ability to undergo chemisorption interaction with the surface of mineral additive particles. For mineral additives of an acidic nature, cation-active surfactants are the most effective, while for basic additives, anion-active ones. Considering that concrete fines predominantly have alkaline reaction and are basic by composition, anion-active surfactants are assumed to be more effective for them. These include traditional naphthalene- and melamine-formaldehyde-based superplasticizers, as well as the latest generation of superplasticizers—polyacrylate and polycarboxylate types.

According to several studies [20,21,22], sodium silicofluoride facilitates the complex alkaline–fluoride activation of mineral additives, thereby increasing their reactivity. Therefore, it was used for the activation of recycled concrete fines.

Based on the reference data, the recycled concrete fines exhibit the following peculiarities, as described in Table 1.

Despite the considerable number of studies on the use of RCF, no research has been carried out on the comparative quantitative assessment of the influence of the main technological factors, the methods of RCF activation, and their interactions. Such an assessment is possible through the development of appropriate multifactor mathematical models.

The current study aimed to investigate the influence of a set of processing and mix design factors and their interactions on the effectiveness of applying recycled concrete fines (RCF) into heavyweight concrete in terms of water demand and compressive strength, and, based on the obtained experimental models, to propose a calculation method for the concrete mix design.

## 2. Materials and Methods of Research

### 2.1. Materials

**Figure 1:**
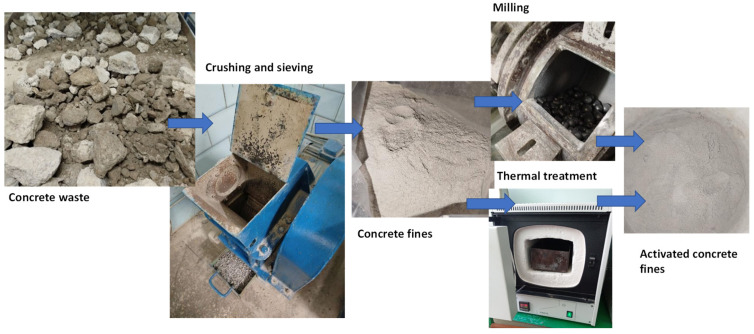
Manufacturing the recycled concrete fines in the lab: crushing the concrete rubble in a jaw crusher; sieving and milling in the ball mill (mechanical activation).

The following materials were used for concrete mix:

- Portland cement CEM I 42.5 (VIPCEM, Kyiv, Ukraine) of the following chemical composition (%): SiO_2_—22.47, Al_2_O_3_—5.26, Fe_2_O_3_—4.07, CaO—66.18, MgO—0.64, SO_3_—0.46, MnO—0.29;
- Silica sand with a fineness modulus of 2.1 and 1.9% of silt and dusty particles;
- Granite crushed stone with a particle size of 5–20 mm;
- Superplasticizer of polyacrylate type Dynamon SR3 (Mapei, Milan, Italy) (SP);
- Sodium silicofluoride (Na_2_SiF_6_) was applied as a chemical activator;
- Recycled concrete fines (RCF) produced in the lab with a particle size less than 1 mm (Figure 1).

The concrete waste used in this study was obtained from debris landfill sites, accumulating the debris resulting from military actions in the Kyiv region (Ukraine) in 2022. The approximate age of the concrete is about 30–40 years.

### 2.2. Methods of Testing

For determining the strength of the original concrete for production recycled concrete fines, the Ukrainian standards for testing were used [33].

XRD and SEM analyses were applied for chemical composition and morphology determination of hardened concrete. The mineralogical composition of the waste was determined using the Aeris Cement diffractometer.

To visualize the microstructure of the concrete and observe the effect of recycled concrete ultrafine on the characteristics of the cement paste and ITZ, an SEM test was performed.

**Figure 2:**
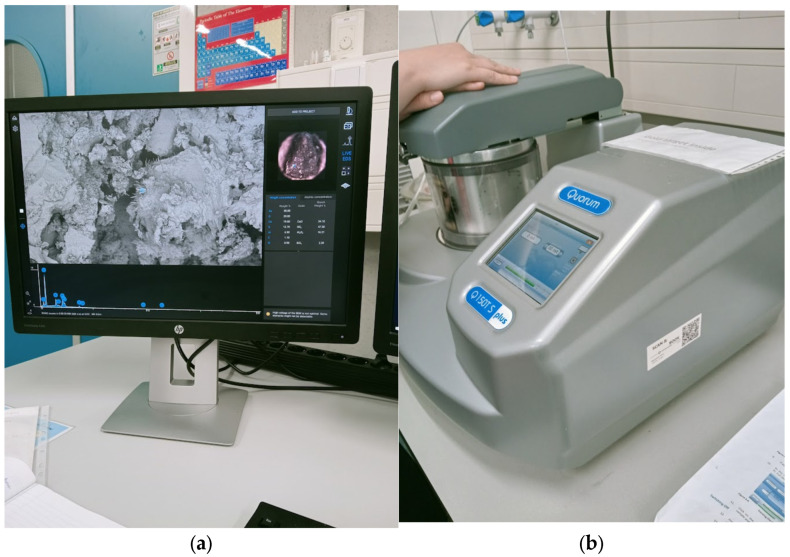
Phenom Pro desktop SEM (**a**) and Quorum 150 T (**b**).

The Phenom Pro desktop SEM for characterization and Quorum 150 T S for preparing the samples where used (Figure 2).

The pozzolanic activity of recycled concrete fines (RCF) was evaluated by measuring the calcium oxide (CaO) uptake from a saturated calcium hydroxide solution in accordance with the Frattini method [34,35]. The specific surface area of the RCF was determined by the Blaine air permeability method [36]. The pH of the RCF suspension was measured using a pH-meter (model OPR-3569) following ASTM E70-19 [37].

The concrete proportioning was conducted using the calculation-experimental method of absolute volumes (ACI Concrete Mix Design), considering the accepted conditions of the experimental plan, ensuring their workability class S3. The compressive strength of the concrete was determined according to EN 12390 [38].

### 2.3. Design of Experiments

The experiments were carried out using the methodology of mathematical experimental design with a three-level, six-factor Box–Behnken B6 plan [39,40], resulting in the derivation of mathematical models of water demand and compressive strength of concrete.

The influence of six factors on the water demand and compressive strength of concrete was investigated. The experimental design conditions are presented in Table 2. The ranges of variation in the thermal treatment temperature of RCF, specific surface, and the content of activating components were determined on the basis of preliminary studies, not included in this paper. All experimental measurements (54 data points) were conducted with controlled repeatability. For each experimental point in the matrix, compressive strength was measured on three replicate cubes of 100 × 100 × 100 mm, with the resulting strength value (f_cm_) expressed as the arithmetic mean of the three measurements. Across the dataset, the typical standard deviation of compressive strength within a single mixture was 0.3–0.8 MPa, corresponding to a coefficient of variation (CV) of approximately 0.5–1.5%, which is consistent with the standard repeatability reported for cement-based materials.

Each water demand (W) value represents the average of two parallel determinations performed to achieve the specified consistency. The typical standard deviation between replicates was 1.5–3.0 L/m^3^, corresponding to CV ≈ 1–2%.

## 3. Results and Discussion

### 3.1. Characterization of Recycled Concrete Fines

**Figure 3:**
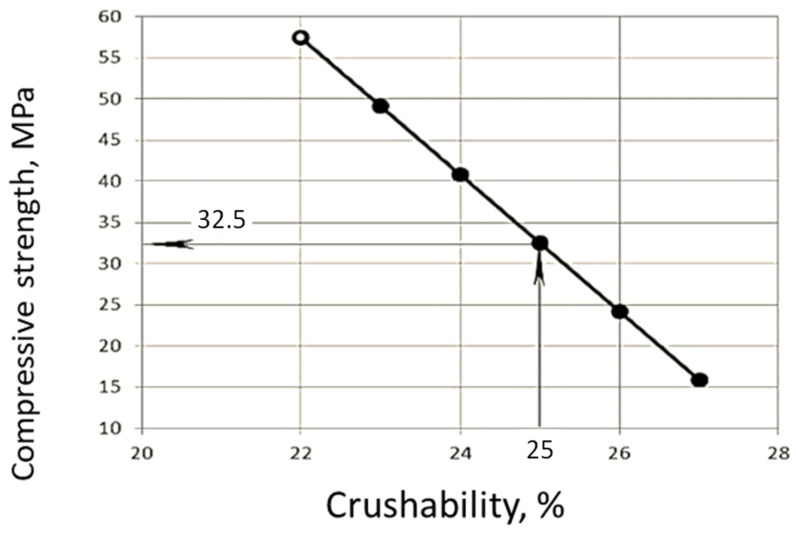
Dependence of the strength of the recycled concrete on its crushability.

Crushability. Using the correlation between the strength and the mass loss after compression tests of the waste in a cylinder according to (Figure 3), the compressive strength of the original concrete from which the waste was obtained was preliminarily estimated.

As it can be seen from Figure 3 the initial strength of the recycled concrete corresponds to strength class C20/25.

The chemical and phase composition of the concrete waste (Table 3) reveals that it was produced on Portland cement, using quartz sand as fine aggregate and granite (or a similar igneous rock) as coarse aggregate. The chemical and phase composition of the waste is consistent with data from different researchers [7,14,24]. This conclusion is supported by the presence of unreacted clinker minerals in the waste, as well as quartz, feldspars, and orthoclase. The amount of clinker minerals indicates the presence of a small portion of unhydrated cement. However, the rather old age of the original concrete is evidenced by the carbonation of calcium hydroxide. It is also confirmed by the relatively low pH value of the suspension of the concrete fines, which increases with the increase in the specific surface (Table 4).

The initial specific surface area of the concrete fines, obtained by crushing the concrete in a jaw crusher, was 130 m^2^/kg (sample #1). By grinding in a laboratory ball mill, it was increased to 250 (sample #2) and 370 m^2^/kg (sample #3) (Figure 2).

For the obtained RCF, in addition to pH, pozzolanic activity was measured (Table 4). As it is seen from the table, increasing the dispersion of the waste as well as its thermal treatment, along with the increase in pH, leads to higher pozzolanic activity of the concrete fines.

### 3.2. Planning of Experiments and Experimental Data

The planning matrix and experimental data are shown in Table 5.

As a result of statistical analysis of the experimental data, adequate regression equations were obtained with a confidence probability of 0.95 for the water demand of the concrete mixture with a slump of 10–12 cm and the compressive strength at ages of 7 and 28 days. The statistical analysis included the following components: a full ANOVA table and a lack-of-fit test. The resulting statistical characteristics are presented in Appendix A. Statistical analysis of the results and construction of graphical dependencies were carried out using the ’Statistica 14.0’ software package [41]. The analysis of the obtained equations shows that all studied factors affect the water demand and compressive strength of concrete with RCF addition, and their influence, can be quantitatively evaluated and ranked (Table 6).

- Water demand

W (L/m^3^) = 188.2 + 4.1·X_1_ + 16.5·X_2_ + 2.8·X_3_ − 27.9·X_4_ + 10 ·X_5_ − 2·X_6_ + 3·X_1_^2^ + 10.3·X_2_^2^ + 2.3·X_3_^2^ − 8·X_4_^2^ + 5.8·X_5_^2^ − 1.7·X_6_^2^ + 2·X_1_·X_2_ + 0.25·X_1_·X_3_ − X_1_·X_4_ + 3·X_1_·X_6_ − 3·X_2_·X_4_ + 3·X_2_·X_5_ − X_2_·X_6_ −0.75·X_3_·X_4_ + 3·X_3_·X_6_ − 5·X_4_·X_5_ (1)

- Compressive strength

f_cm_^7^ (MPa) = 40.9 − 1.4·X_1_ + 9.504·X_2_ + 1.4·X_3_ + 11.0·X_4_ − 4.6·X_5_ + 1.125·X_6_ − 0.56·X_1_^2^ − 0.6·X_2_^2^ − 0.65·X_3_^2^ − 0.35·X_4_^2^ − 0.23·X_5_^2^ − 0.15·X_6_^2^ − 0.33·X_1_X_2_ + 0.81·X_1_·X_3_ + 0.75·X_1_·X_4_ + 0.46·X_1_·X_5_ − 0.15·X_1_·X_6_ + 0.05·X_2_X_3_ − 0.025·X_2_·X_4_ + 0.37·X_2_·X_5_ − 0.3·X_3_·X_4_ + 0.08·X_3_X_5_ + 0.61·X_3_·X_6_ + 1.37·X_4_·X_5_ + 0.8·X_4_·X_6_ (2)

f_cm_^28^ (MPa) = 53.1 + 0.14·X_1_ + 12.1·X_2_ + 2.75·X_3_ + 13.6·X_4_ − 5.2·X_5_ + 2.26·X_6_ − 2.25·X_1_^2^ − 2.7·X_2_^2^ − 1.46·X_3_^2^ −0.69·X_4_^2^ − 2.18·X_5_^2^ + 1.04·X_6_^2^ − 1.15·X_1_·X_2_ + 2.58·X_1_·X_3_ + 2.18·X_1_·X_4_ + 1.3·X_1_·X_5_ − 0.3·X_1_·X_6_ + 0.1·X_2_·X_3_ − 0.05·X_2_·X_4_ + 0.4·X_2_·X_5_ − 0.74·X_3_·X_4_ + 0.1·X_3_·X_5_ − 0.06·X_3_·X_6_ + 1.8·X_4_·X_5_ − 0.54·X_4_·X_6_ (3)

**Figure 4:**
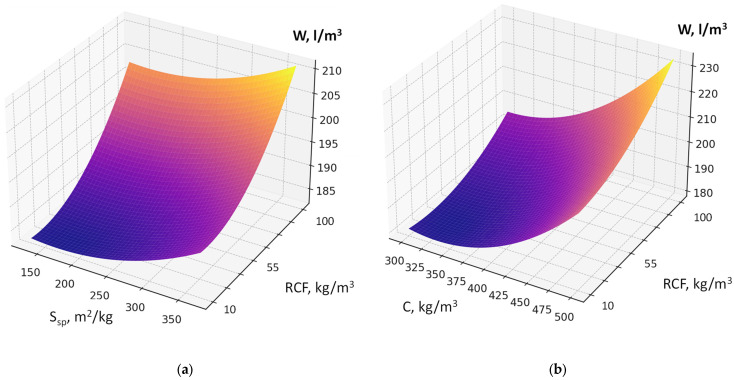
Response surfaces of water demand (W, L/m^3^) of the concrete mixture as a function of RCF content and its specific surface area (S_sp_) (**a**) and cement consumption (C) (**b**).

Recycled concrete fines dosage, if its interaction with other factors is not considered, increases water demand due to its porosity and the presence of amorphized cement hydration products (Figure 4a,b).

**Figure 5:**
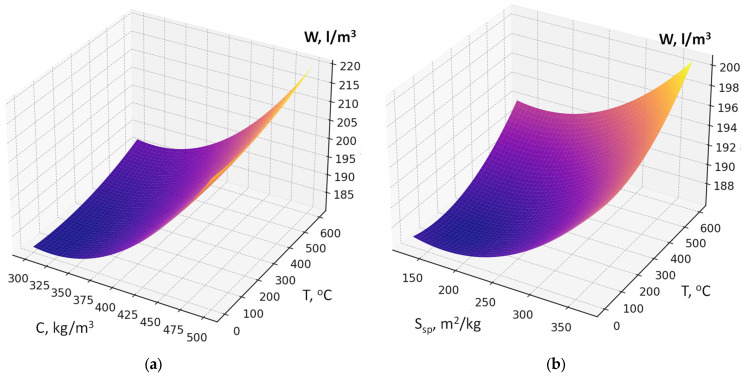
Response surfaces of water demand as a function of cement consumption (C) (**a**), specific surface area of RCF S_sp_, and its thermal treatment temperature (T) (**b**).

The factors responsible for RCF activation (specific surface area, thermal treatment temperature; dosage of chemical activator Na_2_SiF_6_) also affect water demand, although their impact is comparatively less pronounced (Figure 5). An increase in factor X_2_ (cement consumption) causes the maximum rise in water demand of the fresh concrete within the studied range. The presence of a significant quadratic effect of this factor indicates that the highest increase in water demand is observed at cement consumption levels above 400 kg/m^3^ (Figure 5a,b). It confirms the rule of constant water demand of concrete mixtures [26].

**Figure 6:**
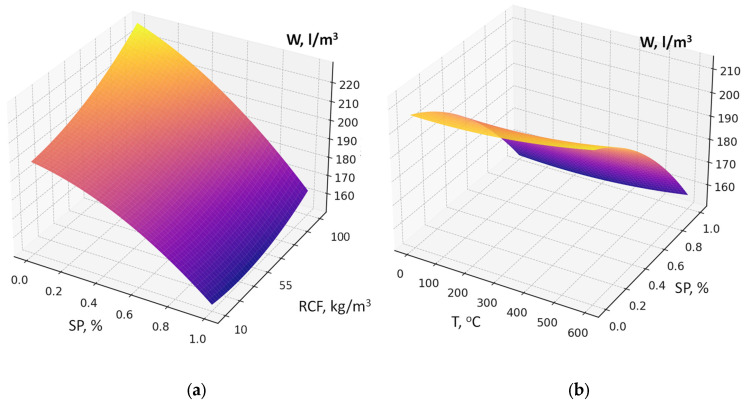
Response surfaces of water demand (W, L/m^3^) as a function of superplasticizer (SP) dosage, RCF content (**a**), and RCF thermal treatment temperature (**b**).

The maximum reduction in water demand is caused by factor X_4_ (superplasticizer SP dosage) (Figure 6a,b). The higher the SP content is, the less water is required to achieve the normal consistency. This factor is characterized by the most significant negative interactions in the model (X_4_ × X_1_, X_4_ × X_2_, X_4_ × X_3_, X_4_ × X_5_): SP effectively compensates the increased water demand caused by other factors (Figure 6a). The increasing of RCF specific surface area (S_sp_, X_1_) also increases water demand by 2–5 L/m^3^, since fine particles require more water for wetting and dispersion in the cement matrix. When combined with high cement content, the increase in water demand reaches 10–15 L/m^3^. The thermal treatment temperature of RCF (X_3_) leads to a relatively small increase in water demand, which may be due to surface activation and the formation of more hydrophilic and reactive phases (2–10 L/m^3^) (Figure 6b). Factor X_6_ (Na_2_SiF_6_ content) causes a slight decrease in water demand within the studied range due to a certain plasticizing effect (5–15 L/m^3^).

An important feature of this model is the presence of significant factor interaction coefficients, the consideration of which makes it possible to draw several important conclusions. The interaction of the amount of RCF and superplasticizer clearly shows that increasing SP dosage almost completely neutralizes the additional water demand caused by an increase in RCF content (Figure 6a): the increase in water demand of 25–35 L/m^3^ is reduced to 5–10 L/m^3^ or less. The interactions of SP with RCF specific surface area (S_sp_) and RCF thermal treatment temperature (T) are negative with respect to their influence on W: with increasing SP content, the negative effects of increased dispersion or thermal treatment are significantly weakened (Figure 6b). Thus, to minimize water demand at high RCF content, especially at elevated cement consumption, the key solution is to increase the SP dosage (reducing water demand by about 50 ± 10 L/m^3^).

**Figure 7:**
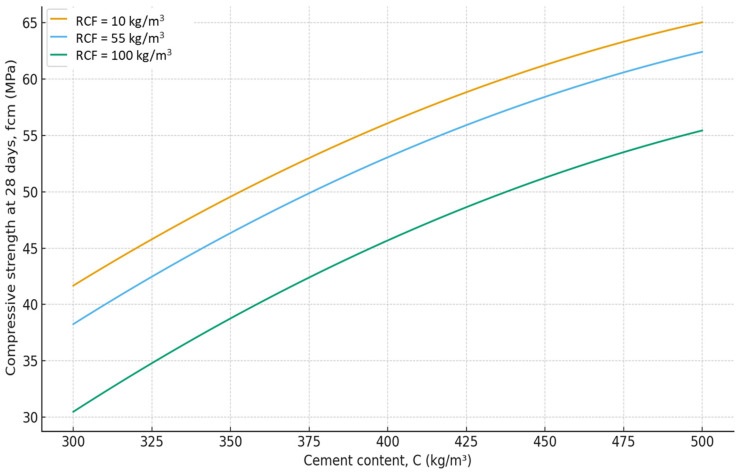
Effect of cement consumption (C) at different levels of recycled concrete fines content on compressive strength of concrete at 28 days. Other factors are fixed at their mid-levels (Table 3).

**Figure 8:**
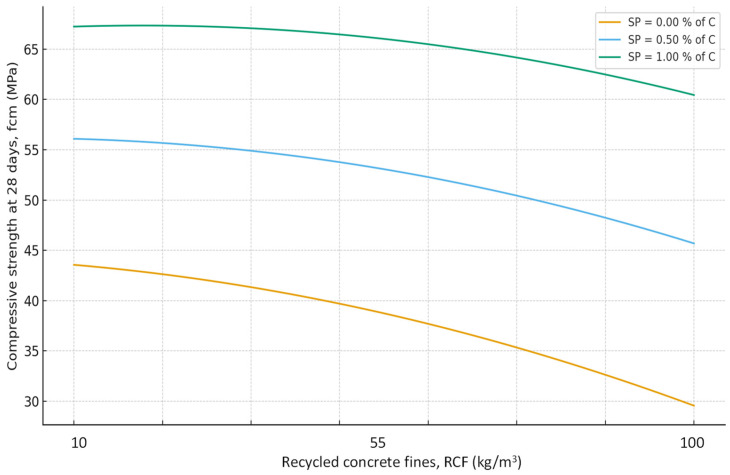
Effect of RCF content and SP dosage on compressive strength of concrete at 28 days. Other factors are fixed at their mid-levels (Table 3).

**Figure 9:**
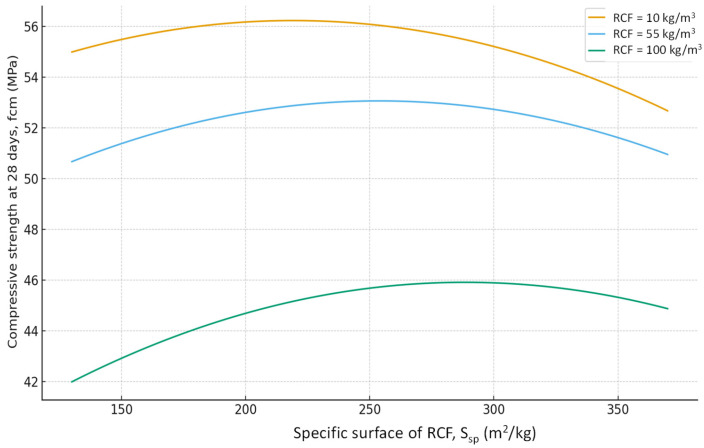
Effect of specific surface area of RCF (S_sp_) at different levels of recycled concrete fines content on compressive strength of concrete at 28 days. Other factors are fixed at their mid-levels (Table 3).

**Figure 10:**
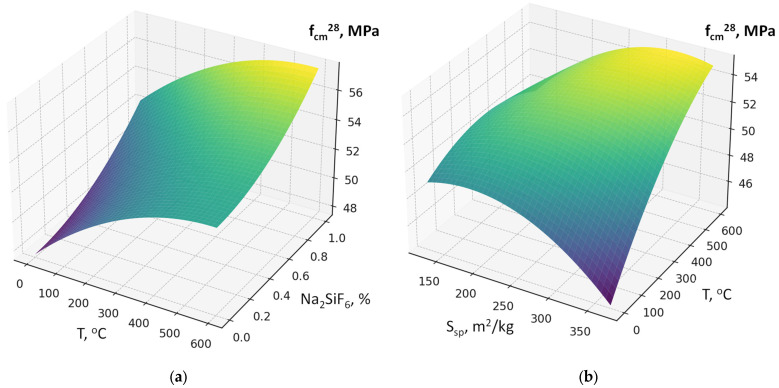
Response surfaces of compressive strength (f_cm_^28^) depending on RCF activation parameters: (**a**) thermal treatment temperature (T) and chemical activator dosage (Na_2_SiF_6_); (**b**) specific surface area (S_sp_).

**Figure 11:**
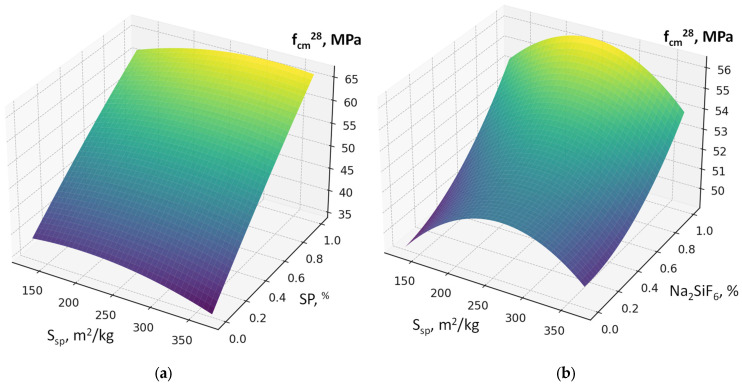
Response surfaces of compressive strength (f_cm_^28^) depending on: (**a**) S_sp_ and SP; (**b**) SP and Na_2_SiF_6_.

**Figure 12:**
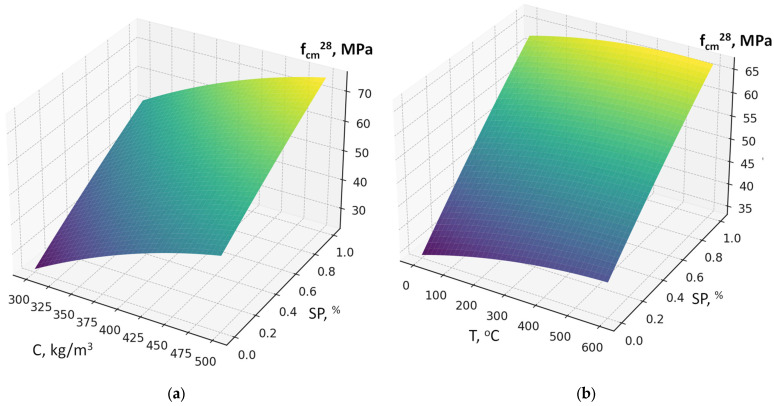
Response surfaces of compressive strength (f_cm_^28^) depending on: (**a**) cement consumption (C) and SP dosage; (**b**) RCF thermal treatment temperature (T).

The six-factor compressive strength model (2) demonstrated high adequacy (R^2^ ≈ 0.991, average error ≈ 1.8 MPa). This allows not only qualitative but also quantitative evaluation of the influence of individual parameters. According to the model (equation), the 28-day compressive strength of concrete varies over a relatively wide range of 20–70 MPa, attributable to significant variations in the w/c ratio (0.35–0.68) (Figure 7, Figure 8, Figure 9, Figure 10, Figure 11 and Figure 12).

As expected, the most significant influence on strength is exerted by factors X_2_ and X_4_ (cement content and SP). An increase in cement consumption and, accordingly, the cement-to-water ratio from the minimum to the maximum level results in a strength gain of approximately 24 MPa (42–45% relative to the average), whereas an equivalent increase in superplasticizer dosage yields approximately 27 MPa (50–55%). At the same time, an increase in the RCF dosage is detrimental: increasing it from the minimum to the maximum level reduces strength by approximately 8–10 MPa (16–18%) (Figure 7). The effect of different RCF activation methods on concrete strength is mixed. Increasing the thermal activation temperature has a noticeable effect: the difference between low (non-thermally activated RCF) and high T is approximately 9%. Activation by Na_2_SiF_6_ within the studied range produces a strength gain of about 7%. Increasing the specific surface area of RCF by grinding in a ball mill (S_sp_) shows an ambiguous effect, producing the most significant strength increase near the mid-level of variation (250 m^2^/kg); further fineness increases reduce strength due to higher water demand (Figure 8).

Interactions between factors confirm that the maximum efficiency of using the RCF as a mineral additive is achieved through combined effects. The most important is the interaction between RCF and SP (X_5_ and X_4_) (Figure 8): if SP is not used, increasing RCF content reduces strength on average by 10–12 MPa, but with sufficient SP dosage (up to 1%) the losses decrease to 3–5 MPa, i.e., they are almost fully compensated. The interaction between Ssp and SP is also positive: with SP present, the effect of mechanical activation of RCF nearly doubles (Figure 9). The combination of mechanical and thermal activation (factors X_1_ and X_3_) (Figure 10b) allows an additional strength gain of up to 4 MPa compared with the simple sum of their effects. Other interactions (e.g., C × SP or T × Na_2_SiF_6_) are less significant (within 1–2 MPa) (Figure 11 and Figure 12).

Thus, the dispersed fraction of recycled concrete in the form of concrete powder generally reduces strength by 15–20%. Still, this effect is significantly offset by SP and additional activation. Key interactions (Ssp × T, Ssp × SP, SP × RCF) demonstrate that the mechanochemical activation of RCF is most effective only when combined with thermal treatment and plasticization.

The above-described tendencies in factor influence on 28-day strength are generally preserved at earlier curing ages (3 and 7 days), although the adverse effect of RCF addition is comparatively more substantial.

The models developed for the strength and water demand of concrete with dispersed fractions of recycled concrete enable the prediction of concrete properties and the determination of mix composition for a given target strength.

**Figure 13:**
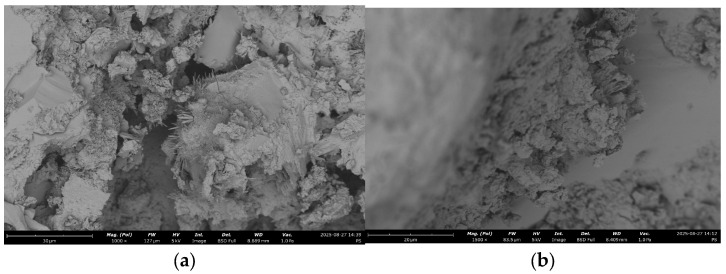

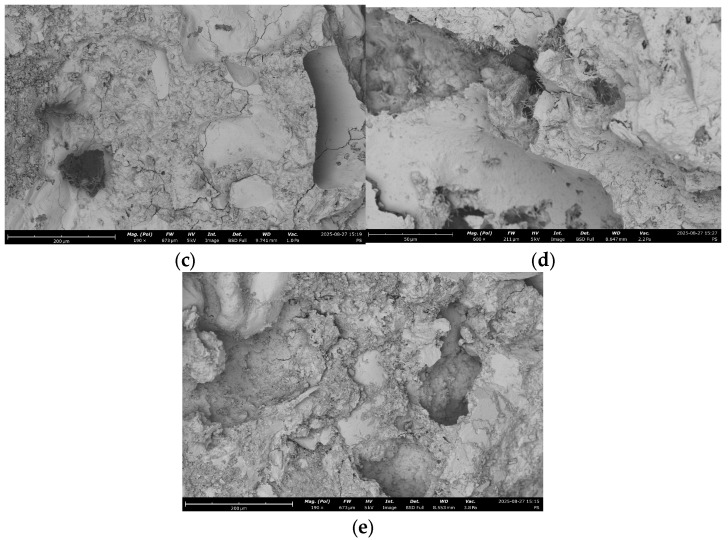
SEM micrograph of concrete microstructure: (**a**) 1; (**b**) 2; (**c**) 3; (**d**) 4; (**e**) 5 (according to Table 4).

SEM images of concrete for the five samples were analyzed (Figure 13):

1. Control;
2. 20% recycled concrete fines;
3. 20% recycled concrete fines + Na_2_SiF_6_ (chemical activation);
4. 20% recycled concrete fines (ground/nonground = 50/50);
5. 20% recycled concrete fines (ground + thermally treated).

The key idea is to determine the effects of RCF on microstructure, chemistry, reactivity, and mechanical performance, and to examine how mechanical and thermal treatments modify these effects relative to a control concrete.

It was expected that RCF would reduce Ca/Si in the paste as C increases, slightly reduce early strength relative to the control, but improve late-age strength due to filler/pozzolanic effects (nucleation).

Chemical activation with Na_2_SiF_6_ is expected to increase reactive Si/Al availability (i.e., higher amorphous content), thereby accelerating pozzolanic reactions and improving strength and CH consumption relative to untreated RCF.

Mechanical grinding was expected to increase surface area and early reactivity but did not change bulk chemistry; the 50/50 mix yields a heterogeneous microstructure and intermediate performance.

As it can be seen in the micrographs, RCF improves the continuity between aggregate and paste phases. Concrete fines serve mainly as fine filler, improving particle packing.

The SEM-EDS analysis presented in Table 7 reveals distinct microstructural and compositional differences between the control concrete and those modified with recycled concrete fines (RCF), with or without additional treatment. The key outcomes reflect the influence of RCF on the calcium-silicate-hydrate (C–S–H) chemistry, degree of carbonation, and presence of other hydration or filler phases.

As it can be seen from Table 7, for control concrete Ca/Si ratio is the highest among all the samples tested as there is higher portlandite content (CH) from full cement hydration. There is also a low level of carbonation since the matrix is denser, with lower open porosity (especially if well cured), and less CO_2_ can diffuse inside.

The control concrete sample exhibits the highest Ca/Si ratio (≈2.8) and Al/Si (≈3.4) among all samples, suggesting the dominance of portlandite (Ca(OH)_2_) and potentially aluminate-rich phases (e.g., AFm/AFt or hydrogarnet). These values are much higher than those typical of C–S–H gel (Ca/Si ≈ 1.2–1.8), implying limited pozzolanic activity and a fully hydrated cement system.

The low carbon content (5.2%) indicates minimal carbonation, consistent with a dense, well-cured microstructure with limited CO_2_ ingress. The microstructure is likely dominated by unreacted clinker phases, portlandite, and conventional C–S–H, with minimal influence from recycled materials.

The inclusion of 20% RCF for sample 2 leads to a substantial drop in Ca/Si (≈1.1) and Al/Si (≈0.4), which may reflect the formation of a low-Ca C–S–H or C–A–S–H phase. The high carbon content (16%) strongly suggests the presence of pre-carbonated fines from the RCF or increased carbonation.

Sample 3 with chemically activated RCF with Na_2_SiF_6_ results in a similar Ca/Si (≈1.1) as untreated RCF, but with lower Al/Si (≈0.2) and a reduced carbon content (4.8%). The activator appears to enhance silica dissolution, thereby promoting the formation of Si-rich C–A–S–H gels with higher density and reduced carbonation. This aligns with expectations for chemically assisted pozzolanic systems, in which lower Ca/Si ratios are associated with more durable C–S–H structures. The reduced carbon content supports the conclusion that chemical treatment helps densify the microstructure and limits CO_2_ penetration.

Sample 4 presents a moderate Ca/Si ratio (≈1.5), with relatively high Al/Si (≈0.4) and elevated carbon (12.5%). The values indicate a heterogeneous matrix, likely due to the coexistence of ground and unground RCF particles.

For sample 5, thermal treatment can decompose portlandite and partially dehydrate C–S–H, resulting in a more silica-rich surface and a lower Ca/Si ratio than in the mechanically treated sample. Al-compounds dehydroxylate/decompose, reducing Al/Si. Carbonates decomposing leads to the reduction of C.

**Figure 14:**
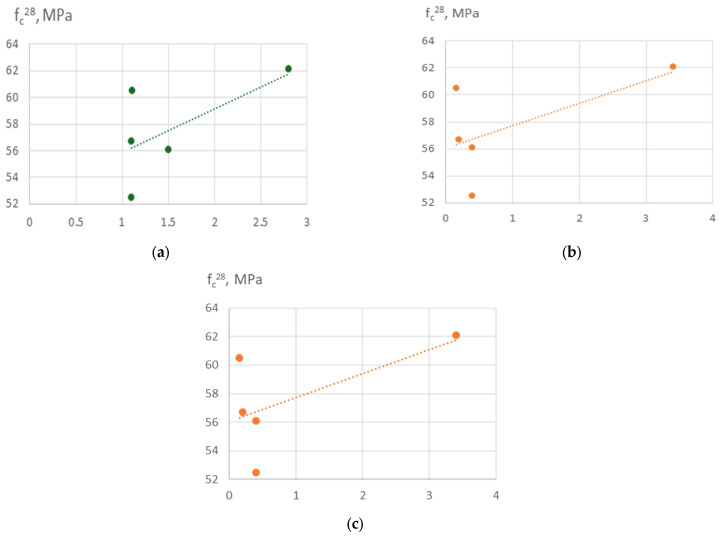
Correlation between compressive strength of concrete samples at the age of 28 days and Ca/Si ratio (**a**), Al/Si (**b**) and C,% (**c**).

The increasing strength of the Ca/Si system (Figure 14a) may be explained by: (i) an increasing amount of calcium reacting with silicates from recycled fines; (ii) a denser microstructure, due to calcium-rich C-S-H filling voids between recycled fines and aggregates more effectively; and (iii) improved bonding between recycled fines and the new paste.

Increasing Al/Si (Figure 14b) in concrete enhances compressive strength because aluminum substitutes into C-S-H to form cross-linked C-A-S-H, densifying the microstructure and improving bonding between matrix and aggregates/fines. Essentially, Al acts like a ‘cross-linker’ in the cement gel network.

Increased carbon content (Figure 14c) reduces the reactivity of the RCF, increases porosity, and interferes with hydration. It results in a weaker microstructure and lower compressive strength.

The results of SEM/EDS tests are consistent with the data reported in [10,42,43].

## 4. Case Study of Concrete Proportioning

The method of concrete proportioning with recycled crushed concrete fines (RCF) as a mineral additive consists of the following steps:

1. For a given 28-day compressive strength, and for a specified amount of RCF chosen based on the required RCF content, the regression Equation (3) is used to determine the necessary dosage of superplasticizer and activation parameters (specific surface area, thermal treatment temperature, and chemical activator dosage) from the standpoint of minimum cost. This task can be solved using multicriteria optimization with local refinement, implemented in the available software (e.g., Microsoft Excel Solver (Microsoft Excel for Microsoft 365, Microsoft Corp., Redmond, WA, USA), MATLAB (R2023b, MathWorks Inc., Natick, MA, USA)).
2. For the parameters obtained from Equation (3), the water demand is necessary to achieve a concrete slump class S3 determined by substituting the obtained values into Equation (1).
3. Considering the selected cement, water, and RCF dosages, the consumption of aggregates is calculated from the equation of absolute volumes:
  (4) $$A=\left(1-\frac{C}{\rho_{c}}-\frac{CF}{\rho_{CF}}-W\right)\rho_{a}$$
4. Finally, the dosages of SP and Na_2_SiF_6_ are determined:
  (5) $${SP}_{c}=C\times SP/100$$
  (6) $${\left({Na}_{2}{SiF}_{6}\right)}_{c}=CF\times \frac{{Na}_{2}{SiF}_{6}}{100}$$

**Prerequisites**

*Mix design of concrete that incorporates recycled concrete fines (RCF) as a mineral additive and determines the necessary activation parameters for RCF while minimizing cement consumption without increasing the overall cost of concrete. The target class of concrete is C25/30, with a slump class S3 (slump 10…15 cm). RCF content is set at 100 kg/m^3^. The real densities of the components are cement—3100 kg/m^3^, RCF—2700 kg/m^3^, aggregates—2650 kg/m^3^.*

*For the cost calculations, the following data are used:*

*Portland cement: 120 €/t*

*RCF: 10 €/t*

*SP: 2.0 €/kg*

*Na_2_SiF_6_ 1.5 €/kg*

*RCF (thermal activation): 2.5 €/50 kg*

*RCF (mechanical activation): 0.50 €/50 kg*

**Steps:**

For a compressive strength of 39.1 MPa, corresponding to concrete class C25/30 with RCF content of 100 kg/m^3^, Equation (3) gives a minimum cost of 43.6 €/m^3^ when the following parameters are applied: SP content—0.88% (of cement mass), specific surface area—252 m^2^/kg, Na_2_SiF_6_ dosage—0.9% (of RCF mass), and no thermal treatment required. The solution was obtained using multicriteria optimization with local refinement in MS Excel “Solver.” Cement consumption—302 kg/m^3^.

For the parameters established in step 1, the water demand necessary to achieve a slump of 10–15 cm, determined from Equation (1), is:

W = 164 L/m^3^.

Considering cement, water, and RCF dosages, the aggregate content (A) is calculated using the absolute volume equation:

$$A=\left(1-\frac{302}{3100}-\frac{100}{2700}-164\right)2650=1859\;\mathrm{kg}/m^{3}.$$

Considering that the proportion of sand in the aggregate mixture is r = 0.32 (by volume), we determine the consumption of sand (S) and crushed stone (CS):

$$S=\left(\frac{A}{\rho_{a}}r\right)\rho_{s}=\left(\frac{1859}{2650}0.32\right)2650=599\;\mathrm{kg}/m^{3};$$

$$CS=\left(\frac{A}{\rho_{a}}(1-r)\right)\rho_{cs}=\left(\frac{1859}{2650}0.68\right)2650=1264\;\mathrm{kg}/m^{3}$$

where *ρ_a_*, *ρ_s_*, *ρ_cs_*—the real density of the aggregate mixture, sand, and crushed stone, respectively.

The SP and Na_2_SiF_6_ dosages are then determined.

$${SP}_{c}=302\times \frac{0.88}{100}=2.65\mathrm{kg}/m^{3}$$

$${\left({Na}_{2}{SiF}_{6}\right)}_{c}=80\times \frac{0.9}{100}=0.72\mathrm{kg}/m^{3}$$

Thus, the calculated nominal composition of the concrete mixture is as follows:

C = 302 kg/m^3^, W=164 L/m^3^, S = 599 kg/m^3^, CS = 1264 kg/m^3^ RCF = 100 kg/m^3^, SP = 2.65 kg/m^3^, Na_2_SiF_6_ = 0.72 kg/m^3^.

The results of multiparametric optimization of concrete mixes containing different amounts of RCF, from the standpoint of minimum cost, are presented in Table 8. The data in Table 8 show that for the specified strength of concrete 50–60 MPa, the use of RCF in the amount of 50–75 kg/m^3^ makes it possible, while maintaining the cost of concrete, to reduce cement consumption by 18–30 kg/m^3^.

## 5. Conclusions

1. By applying a set of mechanical, chemical, thermal and physico-chemical combined activation methods, the quality parameters of concrete rubble and recycled concrete fines (RCF) derived from it were determined, including the strength of the original concrete, chemical and phase composition, specific surface area, and pozzolanic activity.
2. The pozzolanic activity of RCF increases with increasing specific surface area of the particles.
3. Using the methodology of experimental design, six-factor experimental-statistical models were obtained for the water demand and compressive strength of concrete, taking into account cement consumption, RCF content and fineness, superplasticizer dosage, accelerator content (sodium silicofluoride), and RF thermal treatment temperature.
4. Analysis of the models allowed quantitative evaluation of the influence of the studied factors, ranking them by their effect on water demand and strength, and identifying significant interaction effects. The SEM-EDS results provide evidence that the use of RCF, particularly with chemical treatment, can significantly alter the hydration chemistry and microstructure of concrete. The formation of low-Ca, Al-rich C–A–S–H phases improves durability potential, while chemical activation helps suppress carbonation and refine the matrix. Conversely, untreated or mechanically treated RCF introduces greater heterogeneity. These findings support the use of the combined activation methods as a more effective strategy for the application of RCFC in cement and concrete.
5. It was established that the increase in water demand and reduction in compressive strength caused by RCF addition can be mitigated by using a polyacrylate superplasticizer and by RCF activation through thermal treatment combined with sodium silicofluoride. At a cement consumption of 300 kg/m^3^, RCF can be added up to 25%; at 400 kg/m^3^, up to 20%; and at 500 kg/m^3^, up to 15%, yielding comparable compressive strengths.
6. The recommended ranges of fine fractions of recycled concrete (RCF) established in this study are consistent with the general provisions of existing concrete standards (e.g., EN 206 [44], ACI 318 [45]/ACI 555 [46]), which permit the use of mineral admixtures provided that the required performance criteria are met. Accordingly, RCF can be practically classified and utilized as an active mineral admixture without violating current standard specifications for structural concrete.
7. Based on analysis of the models for water demand and compressive strength, and using appropriate software, a method of optimal concrete proportioning with the addition of the recycled concrete has been proposed.
8. The multiparametric optimization of concrete mixtures with RCF, carried out using the developed methodology from the standpoint of minimum cost, indicates that for a target strength of 50–60 MPa, the incorporation of RCF in the amount of 50–75 kg/m^3^ allows a reduction in cement consumption by 18–30 kg/m^3^ while maintaining the overall cost of concrete.

## Figures and Tables

**Table 1:** Characteristics of recycled concrete fines (RCF) and their influence on concrete properties).

Category	Feature	Description	References
Particle morphology of RCFs	Irregular, angular particle shape	RCF after mechanical treatment (crushing and milling) exhibits a rough surface	[23]
	High surface area of particles	Increasing the specific surface area, more reactive sites that can promote pozzolanic and nucleation activity	[24]
	Heterogeneous composition	Particles may contain unhydrated cement grains, calcium hydroxide (portlandite), and C–S–H, calcium carbonate	[25]
Matrix Densification	Changes in ITZ density	More compact microstructure and a denser ITZ in the concrete with RCF compared to a conventional one	[26,27]
	Pore refinement	Ultrafines may fill microvoids and capillary pores, reducing the overall porosity of the cement matrix	[27]
Reaction products	Secondary hydration products	Secondary calcium silicate hydrate (C–S–H), portlandite (CH), ettringite (AFt), and AFm phases.	[28]
	c	Mainly CaCO_3_ formed from aged concrete	[29]
	Unhydrated cement clinker phases	C_3_S, C_2_S, C_3_A, C_4_AF—small but potentially reactive fraction	[7]
	Inert materials	SiO_2_, Al_2_O_3_, Fe_2_O_3_	[30]
ITZ Improvement	Enhanced bonding	ITZ in RCF containing concrete generally shows fewer voids and microcracks compared to control	[26,27]
	Nucleation effect	Ultrafines act as nucleation sites for hydration products, improving the continuity between aggregate and paste phases	[31]
Weakness of the waste	Localized defects	Microcracks or weakly bonded zones, particularly at high replacement ratios, where excess fines can disrupt hydration balance	[30]
	Inert phases	Ultrafine fractions may include inert or carbonated particles (e.g., CaCO_3_), which do not contribute to reactivity and can limit performance gains	[30,32]

**Table 2:** Experimental design conditions.

Factors		Variation Levels			Variation Interval
**Natural**	Coded	−1	0	+1	
Specific surface area of recycled concrete fines (S_sp_, m^2^/kg)	X_1_	130	250	370	120
Cement consumption (C, kg/m^3^)	X_2_	300	400	500	100
Temperature of thermal treatment of recycled concrete fines, (T, °C),	X_3_	0	300	600	300
Dosage of superplasticizer (% of cement mass), SP	X_4_	0	0.5	1	0.5
Recycled concrete fines dosage (RCF, kg/m^3^)	X_5_	10	55	100	45
Dosage of Na_2_SiF_6_ (as % of RCF mass), Na_2_SiF_6_	X_6_	0	0.5	1	0.5

**Table 3:** Chemical and mineralogical composition of recycled concrete fines.

**Chemical Composition, %**	**CaO**	**SiO_2_**	**Al_2_O_3_**		**Fe_2_O_3_**	**MgO**
	13.2	75.5	6.28		3.56	0.61
**Mineralogical Composition, %**	**Quartz**	**Feldspars**	**Calcite**	**C_3_S**	**C_2_S**	**Hydrated Cement Products**
	43.3	15.5	10.9	0.86	2.09	28.3

**Table 4:** Specific surface area and pozzolanic activity of RCF.

Thermal Treatment	Specific Surface Area of Recycled Concrete Fines, m^2^/kg	pH	Pozzolanic Activity of Recycled Concrete Fines, mg/g
Untreated	130	8.1	40.5
	250	9.2	52.3
	370	10.3	70.4
Heat-treated 600 °C	130	8.3	42.2
	250	9.5	60.1
	370	11.7	78.8

**Table 5:** Box–Behnken B6 planning matrix and experimental data *.

No.	Value of the Factors at Experimental Points						Output Parameters			
							Water Demand (W, L/m^3^)	W/C	Compressive Strength (MPa)	
	X_1_	X_2_	X_3_	X_4_	X_5_	X_6_				
									7 Days (f_cm_^7^)	28 Days (f_cm_^28^)
1	−1	−1	0	−1	0	0	199	0.66	21.0	25.2
2	+1	−1	0	−1	0	0	205	0.68	13.7	20.5
3	−1	+1	0	−1	0	0	234	0.47	38.0	47.6
4	+1	+1	0	−1	0	0	248	0.50	31.1	43.3
5	−1	−1	0	+1	0	0	151	0.50	36.9	46.2
6	+1	−1	0	+1	0	0	153	0.51	38.2	52.7
7	−1	+1	0	+1	0	0	174	0.35	57.6	73.4
8	+1	+1	0	+1	0	0	184	0.37	59.2	70.3
9	0	−1	−1	0	−1	0	180	0.60	29.6	36.8
10	0	+1	−1	0	−1	0	207	0.41	47.9	60.8
11	0	−1	+1	0	−1	0	186	0.62	32.1	42.8
12	0	+1	+1	0	−1	0	213	0.43	50.7	67.2
13	0	−1	−1	0	+1	0	194	0.65	19.7	25.4
14	0	+1	−1	0	+1	0	233	0.47	39.1	51.0
15	0	−1	+1	0	+1	0	200	0.67	22.5	31.8
16	0	+1	+1	0	+1	0	239	0.48	42.2	57.8
17	0	0	−1	−1	0	−1	210	0.53	27.9	34.9
18	0	0	+1	−1	0	−1	211	0.53	28.6	38.9
19	0	0	−1	+1	0	−1	156	0.39	47.5	61.1
20	0	0	+1	+1	0	−1	154	0.39	49.9	67.5
21	0	0	−1	−1	0	+1	200	0.50	29.2	37.5
22	0	0	+1	−1	0	+1	213	0.53	32.3	43.9
23	0	0	−1	+1	0	+1	146	0.37	51.9	66.9
24	0	0	+1	+1	0	+1	156	0.39	56.9	65.0
25	−1	0	0	−1	−1	0	197	0.49	35.9	44.0
26	+1	0	0	−1	−1	0	207	0.52	26.9	36.9
27	−1	0	0	+1	−1	0	153	0.38	50.9	63.8
28	+1	0	0	+1	−1	0	159	0.40	50.4	67.9
29	−1	0	0	−1	+1	0	227	0.57	22.0	27.4
30	+1	0	0	−1	+1	0	237	0.59	16.9	25.5
31	−1	0	0	+1	+1	0	163	0.41	42.5	54.4
32	+1	0	0	+1	+1	0	169	0.42	45.9	63.7
33	0	−1	0	0	−1	−1	180	0.60	30.7	39.6
34	0	+1	0	0	−1	−1	209	0.42	49.1	63.8
35	0	−1	0	0	+1	−1	194	0.65	20.9	28.4
36	0	+1	0	0	+1	−1	235	0.47	40.5	54.2
37	0	−1	0	0	−1	+1	178	0.59	34.8	45.0
38	0	+1	0	0	−1	+1	203	0.41	53.2	69.2
39	0	−1	0	0	+1	+1	192	0.64	25.0	33.8
40	0	+1	0	0	+1	+1	229	0.46	44.6	59.6
41	−1	0	−1	0	0	−1	193	0.48	38.6	46.8
42	+1	0	−1	0	0	−1	195	0.49	32.3	43.4
43	−1	0	+1	0	0	−1	192	0.48	36.3	46.8
44	+1	0	+1	0	0	−1	195	0.49	37.8	53.7
45	−1	0	−1	0	0	+1	177	0.44	41.9	51.6
46	+1	0	−1	0	0	+1	191	0.48	34.8	47.0
47	−1	0	+1	0	0	+1	188	0.47	42.0	54.1
48	+1	0	+1	0	0	+1	203	0.51	42.7	59.8
49	0	0	0	0	0	0	186	0.47	40.6	53.4
50	0	0	0	0	0	0	188	0.47	41.3	52.4
51	0	0	0	0	0	0	187	0.47	40.9	53.7
52	0	0	0	0	0	0	190	0.48	41.0	52.9
53	0	0	0	0	0	0	190	0.48	41.5	53.6
54	0	0	0	0	0	0	188	0.47	40.1	52.4

* In all experimental concrete mixes, the sand fraction in the aggregate mixture was kept constant and amounted to 0.32 (by volume).

**Table 6:** Results of factor ranking in Equations (1)–(3).

Output Parameter	Factors That Increase the Parameter	Factors That Decrease the Parameter
Water demand, L/m^3^	X_2_ > X_5_ > X_1_ > X_3_	X_4_ > X_6_
Compressive strength (7, 28 days)	X_4_ > X_2_ > X_6_ > X_3_	X_5_ > X_1_

**Table 7:** Key outcomes of SEM and EDS tests of concrete.

#	RCF	Treatment	SEM Image with EDS Indicative Point	Ca/Si	Al/Si	C, %
1	0	-	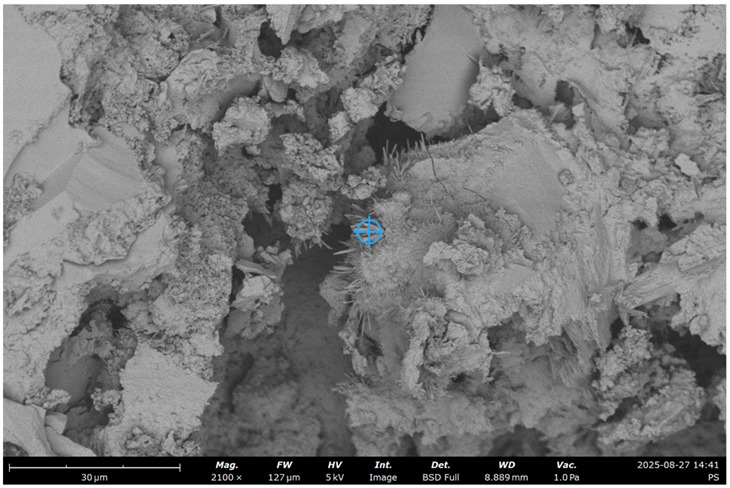	2.8	3.4	5.2
2	20%	-	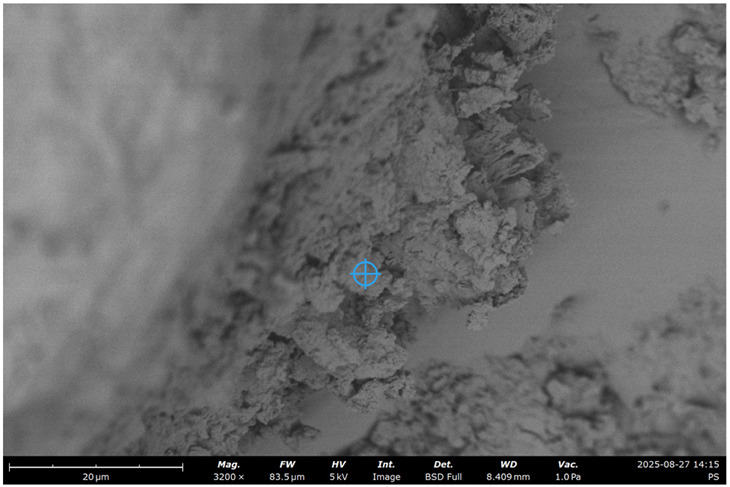	1.1	0.4	16
3	20%	Chemical	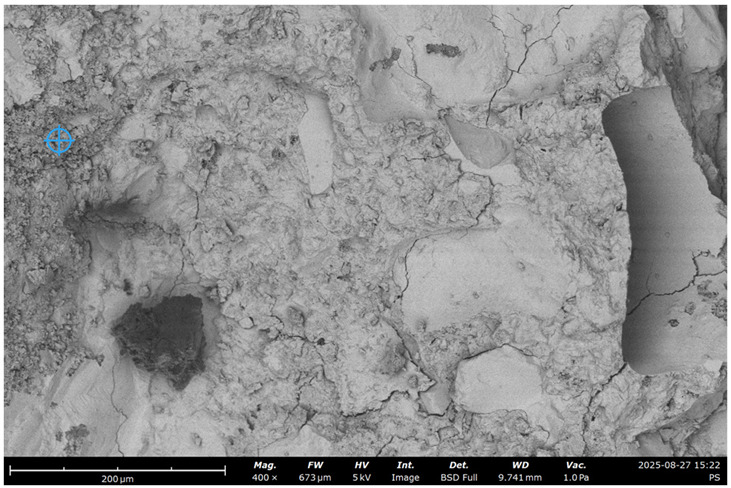	1.1	0.2	4.8
4	20%	Mechanical	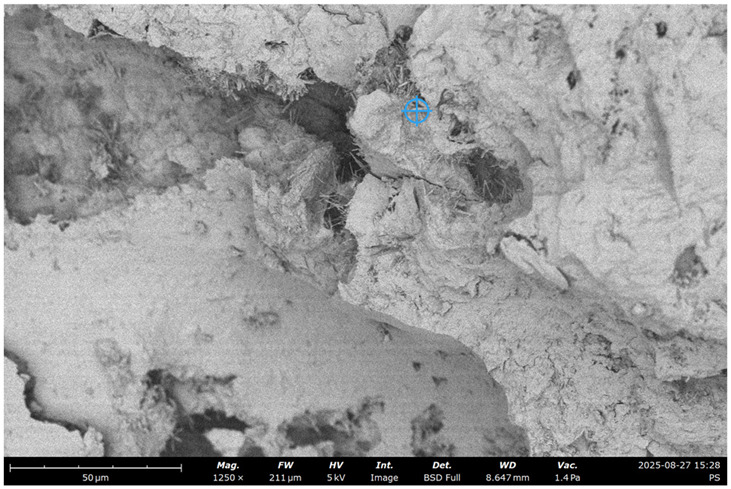	1.5	0.4	12.5
5	20%	Mechanical + thermal	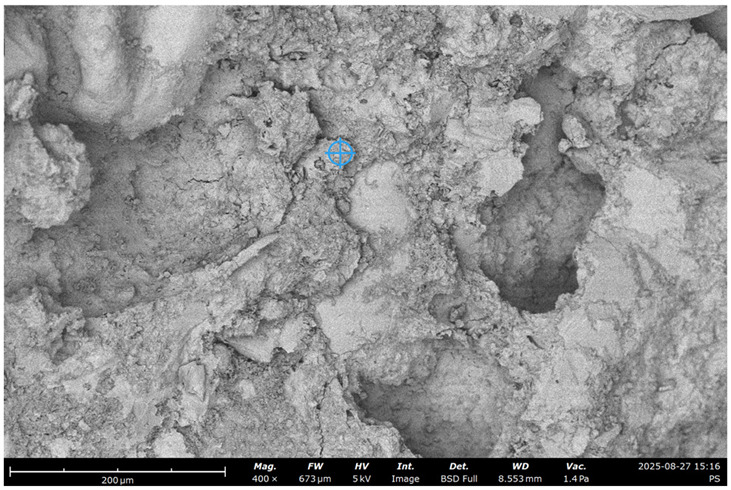	1.1	0.16	2.4

**Table 8:** Results of concrete mix design according to the proposed method for different strength classes.

f_c_^28^ (MPa)	RCF (kg/m^3^)	C (kg/m^3^)	SP (% of C)	W (L/m^3^)	W/C	S_sp_ (m^2^/kg)	T (°C)	Na_2_SiF_6_ (% of RCF)	Cost (EUR/m^3^)
50	100	304.5	0.99	160	0.53	336	330	0.91	47.26
	75	301.9	0.95	156	0.52	331	230	0.77	44.86
	50	301.2	0.93	148	0.49	207	54	0.99	42.93
	25	300.0	0.82	163	0.54	292	245	0.84	41.82
	0	318.5	1.00	162	0.51	130	0	0.00	44.58
60	100	377.2	0.99	162	0.43	321	284	0.92	57.05
	75	351.0	0.97	159	0.45	343	427	0.87	52.92
	50	328.1	0.95	157	0.48	313	524	0.99	48.55
	25	316.9	0.97	160	0.50	334	576	0.99	45.79
	0	384.4	1.00	161	0.42	130	0	0.00	53.8

## Data Availability

The original contributions presented in this study are included in the article. Further inquiries can be directed to the corresponding author.

## Appendix A. Statistical Indicators of the Experimental–Statistical Models

### Appendix A.1. Water Demand (W, Equation (1))

**Table A1:** Full ANOVA table (Equation (1)).

Source	Sum of Squares (SS)	df	F-Value	*p*-Value
X1	400.1667	1.0	752.1205	<0.001
X2	6534.0	1.0	12,280.7711	<0.001
X3	192.6667	1.0	362.1205	<0.001
X4	18,704.1667	1.0	35,154.8193	<0.001
X5	240.0	1.0	4510.8434	<0.001
X6	96.0	1.0	180.4337	<0.001
I(X1 ^ 2)	94.2937	1.0	177.2266	<0.001
I(X2 ^ 2)	1086.5079	1.0	2042.1113	<0.001
I(X3 ^ 2)	53.3651	1.0	100.3006	<0.001
I(X4 ^ 2)	653.7222	1.0	1228.6827	<0.001
I(X5 ^ 2)	343.3651	1.0	645.3609	<0.001
I(X6 ^ 2)	30.5079	1.0	57.3402	<0.001
X1:X2	32.0	1.0	60.1446	<0.001
X1:X3	0.5	1.0	0.9398	0.3413
X1:X4	16.0	1.0	30.0723	<0.001
X1:X5	<0.001	1.0	0.0	1.0
X1:X6	72.0	1.0	135.3253	<0.001
X2:X3	<0.001	1.0	<0.001	1.0
X2:X4	72.0	1.0	135.3253	<0.001
X2:X5	144.0	1.0	270.6506	<0.001
X2:X6	8.0	1.0	15.0361	0.0006
X3:X4	4.5	1.0	8.4578	0.0073
X3:X5	<0.001	1.0	<0.001	1.0
X3:X6	144.0	1.0	270.6506	<0.001
X4:X5	200.0	1.0	375.9036	<0.001
X4:X6	<0.001	1.0	<0.001	1.0
X5:X6	<0.001	1.0	<0.001	1.0
Residual	13.8333	26.0		

Statistical indicators: R^2^ = 0.9996, Adjusted R^2^ = 0.9991. Lack-of-fit: F = 0.0186, *p*-value ≈ 1.0 (model adequate).

### Appendix A.2. Compressive Strength at 7 Days (Equation (2))

**Table A2:** Full ANOVA table (f_cm_^7^, Equation (2)).

Source	Sum of Squares (SS)	Df	F-Value	*p*-Value
X1	47.320	1.0	964.177	4.426 × 10^−22^
X2	2167.90	1.0	44,172.077	1.566 × 10^−43^
X3	47.039	1.0	958.464	4.772 × 10^−22^
X4	2910.603	1.0	59,305.036	3.406 × 10^−45^
X5	507.839	1.0	10,347.499	2.39 × 10^−35^
X6	100.860	1.0	2055.073	2.816 × 10^−26^
I(X1 ^ 2)	47.545	1.0	968.768	4.167 × 10^−22^
I(X2 ^ 2)	35.680	1.0	727.001	1.561 × 10^−20^
I(X3 ^ 2)	13.900	1.0	283.223	1.694 × 10^−15^
I(X4 ^ 2)	0.057	1.0	1.178	0.287
I(X5 ^ 2)	59.245	1.0	1207.161	2.546 × 10^−23^
I(X6 ^ 2)	5.221	1.0	106.392	1.107 × 10^−10^
X1:X2	0.061	1.0	1.248	0.274
X1:X3	30.420	1.0	619.823	1.152 × 10^−19^
X1:X4	72.675	1.0	1480.802	1.878 × 10^−24^
X1:X5	7.605	1.0	154.955	1.851 × 10^−12^
X1:X6	0.320	1.0	6.520	0.0168
X2:X3	0.044	1.0	0.916	0.347
X2:X4	6.661	1.0	135.726	8.052 × 10^−12^
X2:X5	1.322	1.0	26.946	2.031 × 10^−5^
X2:X6	1.037 × 10^−29^	1.0	2.113 × 10^−28^	1.0
X3:X4	1.620	1.0	33.008	4.76 × 10^−6^
X3:X5	0.0449	1.0	0.916	0.347
X3:X6	6.0024	1.0	122.304	2.503 × 10^−11^
X4:X5	15.124	1.0	308.179	6.158 × 10^−16^
X4:X6	5.12	1.0	104.322	1.362 × 10^−10^
X5:X6	5.404 × 10^−29^	1.0	1.101 × 10^−27^	1.0
Residual	1.276	26.0		

Statistical indicators: R^2^ = 0.9998, Adjusted R^2^ = 0.9996.No signs of significant lack-of-fit were detected, *p*-value ≈ 1.0 (model adequate).

### Appendix A.3. Compressive Strength at 28 Days (Equation (3))

**Table A3:** Full ANOVA table (f_cm_^28^, Equation (3)).

Source	Sum of Squares (SS)	Df	F-Value	*p*-Value
X1	49.15	1.0	1080.84	<0.001
X2	2288.68	1.0	47,592.64	<0.001
X3	51.30	1.0	1039.82	<0.001
X4	2935.07	1.0	70,898.75	<0.001
X5	596.69	1.0	10,382.20	<0.001
X6	107.08	1.0	2414.85	<0.001
I(X1 ^ 2)	55.08	1.0	1082.12	<0.001
I(X2 ^ 2)	36.53	1.0	747.75	<0.001
I(X3 ^ 2)	14.89	1.0	290.59	<0.001
I(X4 ^ 2)	0.06	1.0	1.40	0.2880
I(X5 ^ 2)	68.33	1.0	1261.56	<0.001
I(X6 ^ 2)	6.16	1.0	122.37	<0.001
X1:X2	0.07	1.0	1.28	0.3001
X1:X3	35.48	1.0	699.87	<0.001
X1:X4	84.34	1.0	1762.08	<0.001
X1:X5	9.01	1.0	171.78	<0.001
X1:X6	0.35	1.0	7.46	0.0169
X2:X3	0.05	1.0	0.98	0.3692
X2:X4	7.26	1.0	149.41	<0.001
X2:X5	1.33	1.0	31.13	<0.001
X2:X6	<0.001	1.0	0.00	1.0643
X3:X4	1.90	1.0	36.76	<0.001
X3:X5	0.04	1.0	1.09	0.3978
X3:X6	7.16	1.0	126.00	<0.001
X4:X5	15.78	1.0	317.99	<0.001
X4:X6	5.92	1.0	120.34	<0.001
X5:X6	<0.001	1.0	<0.001	1.0000
Residual	1.47	26.0		

Statistical indicators: R^2^ = 0.9908, Adjusted R^2^ = 0.9812. No signs of significant lack-of-fit were detected, *p*-value ≈ 1.0 (model adequate).

To confirm the reliability of the developed mathematical models of water demand and concrete strength, statistical validation was performed using k-fold cross-validation (k = 10). This approach makes it possible to assess the stability of the model beyond the training dataset and minimizes the risk of overfitting. According to the analysis results, for the strength model (28 days), the mean coefficient of determination was R^2^_cv = 0.93, indicating a high ability of the model to reproduce the experimental data. The mean absolute error was MAE = 1.85 MPa, and the root mean square error was RMSE = 2.45 MPa, which confirms the good predictive accuracy of the model within the studied factor space. For the water demand model, the following generalized indicators were obtained: MAE ≈ 3.8 L/m^3^, RMSE ≈ 5.6 L/m^3^, R^2^_cv ≈ 0.94, confirming the stability of the regression parameters and the high generalization capability of the model.
